# The multifaceted role of YAP in the tumor microenvironment and its therapeutic implications in cancer

**DOI:** 10.1038/s12276-025-01551-9

**Published:** 2025-10-01

**Authors:** Hyung-Sik Kim, Jeong-Seok Nam

**Affiliations:** 1 Department of Oral Biochemistry, Dental and Life Science Institute, School of Dentistry, Pusan National University, Yangsan, Republic of Korea; 2 Department of Life Sciences, Gwangju Institute of Science and Technology, Gwangju, Republic of Korea

**Keywords:** Cancer microenvironment, Cancer immunotherapy

## Abstract

The tumor microenvironment (TME) plays a critical role in cancer progression, immune evasion and therapeutic resistance. The transcriptional coactivators YAP and TAZ, key effectors of the Hippo signaling pathway, have emerged as central regulators of TME remodeling. YAP/TAZ are activated in both tumor and stromal compartments, where they function as mechanotransducers and integrate canonical Hippo pathway suppression, noncanonical microenvironmental cues and genetic or epigenetic signals to drive transcriptional programs. These changes collectively facilitate tumor immune evasion. YAP/TAZ further promote angiogenesis and upregulate PD-L1 expression in tumor cells and cancer-associated fibroblasts, and a subset of immunosuppressive cells in the TME, contributing to resistance to ICB. In addition to their tumor-intrinsic and stromal functions, YAP/TAZ impair antitumor immunity by altering immune cell differentiation and dampening effector responses. Targeting the YAP/TAZ–TEAD axis has shown potential efficacy when combined with immune checkpoint inhibitors, chimeric antigen receptor T cell therapies and tumor vaccines. Although challenges such as tumor selectivity and resistance mechanisms persist, advances in single-cell and spatial transcriptomics are enabling the dissection of YAP/TAZ-regulated networks and guiding the development of more precise therapeutic strategies. Collectively, YAP/TAZ inhibition offers a promising avenue to reprogram the TME and enhance the efficacy of next-generation cancer immunotherapies.

## Targeting YAP/TAZ enhances cancer immunotherapy effectiveness

The tumor microenvironment (TME) plays a crucial role in cancer progression and treatment resistance. This review highlights how the Hippo signaling pathway, particularly its effectors YAP and TAZ, influences the TME. YAP/TAZ activation promotes cell proliferation and survival while also reshaping the TME by altering immune cell behavior, inducing fibrosis and angiogenesis, thereby facilitating immune evasion. The review concludes that targeting YAP/TAZ could improve cancer treatments by making tumors more responsive to therapies. Future implications include developing drugs that inhibit YAP/TAZ to enhance the effectiveness of existing cancer treatments, potentially leading to better patient outcomes.

This summary was initially drafted using artificial intelligence, then revised and fact-checked by the author.

## Introduction

The tumor microenvironment (TME) has emerged as a critical determinant of cancer progression, immune evasion and therapeutic resistance. This dynamic and heterogeneous ecosystem comprises not only malignant tumor cells but also a variety of nonmalignant components, including immune cells, cancer-associated fibroblasts (CAFs), endothelial cells, the extracellular matrix (ECM) and various soluble factors such as cytokines and chemokines^1^. Tumor−TME interactions are reciprocal and context dependent, with each component influencing the behavior of the other. The TME supports tumor growth by providing essential nutrients and growth signals, facilitating invasion and metastasis through ECM remodeling, and promoting drug resistance by establishing physical barriers and activating prosurvival signaling^2^. Moreover, spatial and cellular heterogeneity within the TME contributes to intratumoral complexity and variable treatment responses. Thus, understanding the TME is essential for designing therapies that target both cancer cells and their surrounding immunosuppressive, tumor-supportive environment.

**Fig. 1:**
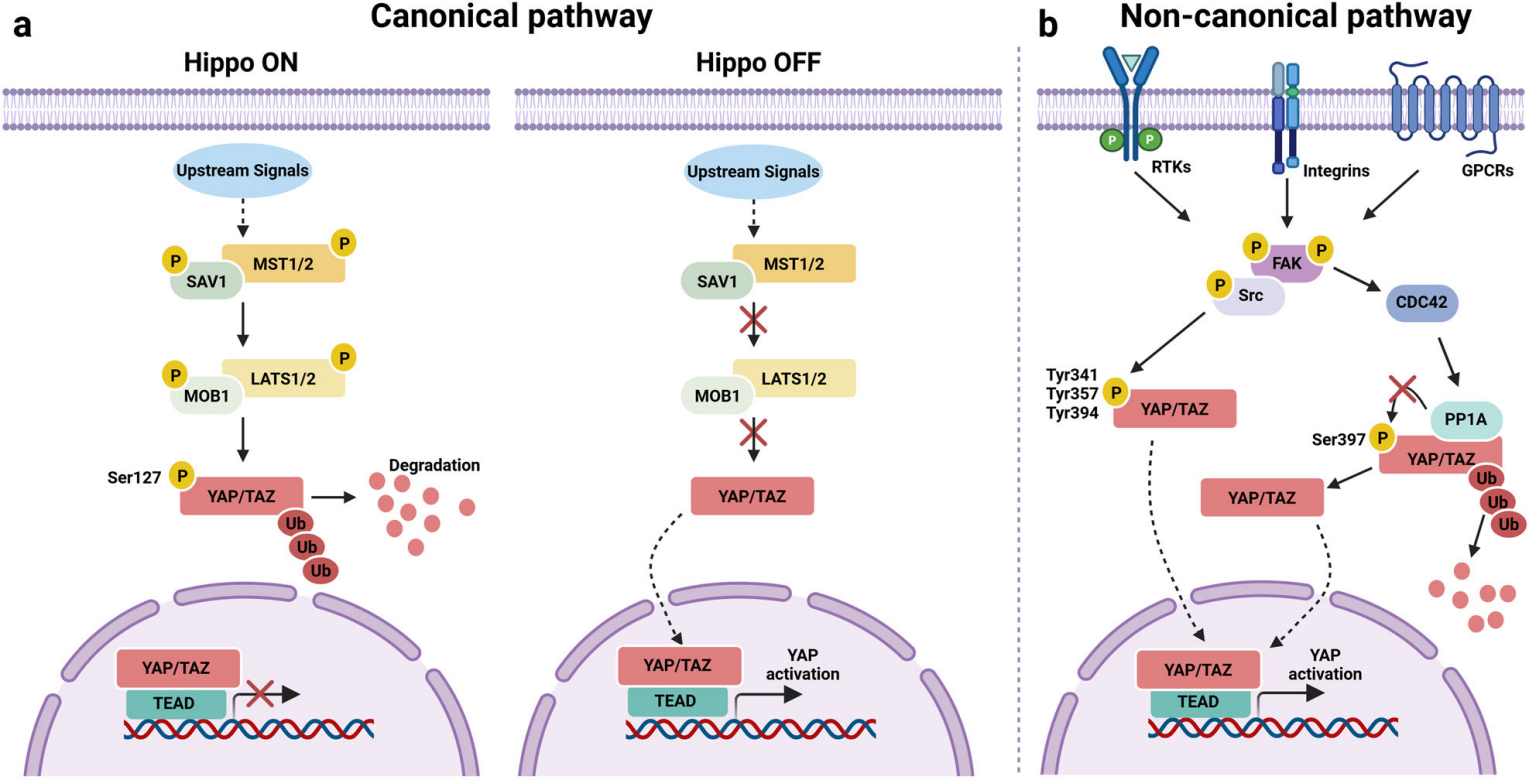
Canonical and noncanonical regulation of YAP/TAZ activity. **a** In the canonical Hippo pathway, when the pathway is ‘ON’, MST1/2 kinases phosphorylate and activate LATS1/2, leading to phosphorylation and cytoplasmic retention of the transcriptional coactivators YAP and TAZ, thereby preventing their nuclear activity. When the Hippo pathway is ‘OFF’, unphosphorylated YAP/TAZ translocate to the nucleus, bind TEAD transcription factors, and drive the expression of target genes. **b** The noncanonical Hippo-independent pathway promotes YAP/TAZ activation via alternative mechanisms, including integrin-mediated FAK–Src signaling. Upon upstream activation, FAK autophosphorylates at Tyr397, creating an SH2-binding site that recruits Src family kinases. Activated FAK and Src reciprocally phosphorylate each other and downstream targets, ultimately promoting YAP activity via two complementary mechanisms: (1) direct Src-mediated tyrosine phosphorylation of YAP, which enhances its nuclear localization and transcriptional function and (2) an integrin-FAK–CDC42–PP1A cascade that removes LATS-added inhibitory phosphates from YAP, facilitating its nuclear accumulation. Created in BioRender. Kim, H. (2025) https://BioRender.com/1vsxkc6.

Among the oncogenic pathways, the Hippo cascade plays a central role in controlling organ size, tissue homeostasis and cell fate. Its core effectors, Yes-associated protein (YAP) and transcriptional coactivator with PDZ-binding motif (TAZ), are key transcriptional coactivators. Under normal conditions, MST1/2 and LATS1/2 kinases, along with scaffold proteins such as SAV1 and MOB1A/B, phosphorylate YAP/TAZ, promoting their cytoplasmic sequestration and degradation^3,4^. Upon Hippo pathway inactivation, YAP/TAZ translocate to the nucleus and interact with TEAD transcription factors to induce genes involved in cell proliferation, survival and stemness (Fig. 1a). While canonical serine phosphorylation restrains their activity, noncanonical modifications—such as tyrosine phosphorylation by Src—can enhance YAP/TAZ nuclear localization and transcriptional output under conditions of cellular stress, DNA damage or inflammation^5^ (Fig. 1b). Persistent YAP/TAZ activation is observed in various cancers and correlates with poor prognosis.

Emerging evidence underscores the pivotal role of YAP and TAZ as key mediators of tumor–TME crosstalk^6^. Beyond their cell-intrinsic roles, YAP/TAZ broadly regulate nonmalignant components of the TME including CAFs, endothelial cells and immune subsets such as T cells and myeloid-derived suppressor cells^6^. These interactions drive fibrosis, immune evasion and angiogenesis, reinforcing a protumorigenic niche. Accordingly, YAP/TAZ have emerged as compelling therapeutic targets, with inhibition of the YAP/TAZ–TEAD axis offering a strategy to reprogram the TME, reinstate antitumor immunity and enhance responses to immunotherapy.

## Yap activation and its impact on the TME

Activation of the transcriptional coactivators YAP and TAZ is a central event regulating cell proliferation, survival, differentiation and tissue homeostasis. Dysregulation of YAP/TAZ is frequently linked to pathological conditions, particularly cancer. Although initially characterized as effectors of the Hippo signaling pathway, YAP/TAZ are now recognized as integrative hubs that respond to a broad spectrum of intracellular signals and extracellular cues. Elucidating how YAP/TAZ become activated is essential to understanding their roles in TME modulation. This section outlines the major mechanisms of YAP/TAZ activation, including Hippo pathway inactivation, mechanical cues, noncanonical microenvironmental signals and genetic or epigenetic alterations.

### Canonical Hippo pathway inactivation

The core mammalian Hippo signaling pathway acts as a conserved tumor suppressor cascade that primarily restricts tissue growth by inhibiting the activity of YAP and TAZ. In its active state, the Hippo pathway leads to inhibitory phosphorylation of YAP/TAZ, preventing their nuclear translocation and subsequent transcriptional activity. Conversely, inactivation of the core Hippo pathway is a major mechanism leading to YAP/TAZ activation. The central components of the mammalian Hippo pathway include MST1/2 and the NDR/LATS family kinases, LATS1 and LATS2. These kinases function within complexes involving scaffold proteins such as SAV1 and MOB1A/B. When the core Hippo pathway is inactive, LATS1/2 are not sufficiently active to phosphorylate YAP/TAZ. In this unphosphorylated state, YAP/TAZ are not targeted for degradation. This allows them to translocate into the nucleus^7^ (Fig. 1a).

Once in the nucleus, YAP/TAZ act as transcriptional coactivators by interacting with DNA-binding transcription factors. Their primary binding partners are the transcriptional enhanced associate domain family members (TEAD1−4). YAP/TAZ lack intrinsic DNA-binding domains, making their interaction with TEADs essential for targeting specific gene promoters and enhancers^8^. In the absence of nuclear YAP/TAZ, TEADs can bind to transcriptional repressors such as VGLL4. The binding of YAP/TAZ to TEADs displaces VGLL4, converting TEADs into transcriptional activators and promoting the expression of genes involved in proliferation, survival, and migration. Beyond TEADs, YAP/TAZ interact with a variety of other transcription factors (for example, AP-1, SMADs, p73, RUNX1/2, ERBB4, PAX3 and TBX5) and transcriptional coactivators or chromatin modifiers (for example, BRD4, ARID1A and CDK7) to regulate gene expression and chromatin structure^9^.

The specific kinases and mechanisms that activate LATS1/2 can vary depending on the cell type and the nature of the activating signal, suggesting flexibility and context specificity within the canonical pathway. For example, TAO kinases (TAOK1/2/3) can phosphorylate and activate MST1/2. MAP4Ks (MAP4K4/6/7) have also been shown to regulate LATS1/2 phosphorylation^8^. These upstream kinases integrate signals from various cellular processes and external cues to modulate the activity of the core Hippo pathway.

### Genetic and epigenetic alterations

YAP/TAZ activity can be constitutively altered by genetic and epigenetic changes, especially in cancer. These alterations may affect YAP/TAZ expression, protein function or the accessibility of their target genes. Although point mutations in core Hippo components are relatively rare, amplification of *YAP1* and *WWTR1* (TAZ) is more frequent in specific cancers, particularly squamous cell carcinoma and ovarian cancer^6^.

Gene fusions involving *YAP1* or *WWTR1*, typically retaining the TEAD-binding domain (TBD), often fuse to transcriptional regulators and evade LATS1/2-mediated phosphorylation, resulting in persistent nuclear localization^10^. While activating point mutations in *YAP1*/*WWTR1* are uncommon, mutations or deletions in upstream negative regulators such as *NF2* or *LATS1/2* can lead to hyperactivation of YAP/TAZ.

Beyond genetic alterations, YAP/TAZ activity is further shaped by epigenetic mechanisms, including DNA/histone modifications and noncoding RNAs^11^. Changes in DNA methylation enhance YAP/TAZ activation and, conversely, YAP/TAZ influence DNA methylation by regulating DNA methyltransferases (DNMTs) and ten-eleven translocation (TET) dioxygenases^12^. For example, YAP induces TET1 to promote local demethylation at its target loci, while TAZ has been shown to recruit DNMT1 to silence negative regulators such as *LATS2* via hypermethylation^13^.

YAP/TAZ interact with chromatin remodeling complexes such as SWI/SNF and NuRD, modulating chromatin accessibility and target gene transcription^14^. These regions are enriched with active histone marks such as H3K4me3 and H3K27ac, reflecting their transcriptional activity^15^.

Noncoding RNAs play crucial roles in regulating YAP/TAZ activity. MicroRNAs bind to the 3′-untranslated region of *YAP1* or *WWTR1* mRNA, leading to translational repression^16^. Long noncoding RNAs (lncRNAs) and circular RNAs act as competing endogenous RNAs, sponging microRNAs that would otherwise inhibit YAP/TAZ^17^. Long noncoding RNAs interact directly with YAP/TAZ proteins or proteins that affect their stability or nuclear translocation^18^. Together, these genetic and epigenetic mechanisms form multilayered feedback loops that fine-tune YAP/TAZ signaling and transcriptional output.

### Mechanical cues

The physical properties of the cellular microenvironment, such as the stiffness of the ECM, cell density and cell shape, are potent regulators of YAP/TAZ activity. YAP/TAZ function as key mechanotransducers, translating these physical stimuli into biochemical signals that regulate cell behavior^19^. Rather than acting as passive modulators, mechanical forces serve as central upstream regulators of YAP/TAZ activity, often overriding canonical Hippo signaling in certain contexts.

Cells perceive mechanical cues through various structures, including cell−ECM adhesions (focal adhesions), cell−cell junctions (adherens junctions or tight junctions) and the actin cytoskeleton. ECM stiffness and cell shape are critical determinants. Cells spread on stiff substrates or large adhesive areas typically exhibit high YAP/TAZ signaling and nuclear localization^19^. Conversely, cells on soft matrices or confined to small areas are more rounded, leading to YAP/TAZ inhibition and cytoplasmic localization. This response is crucial for processes such as stem cell differentiation, where substrate stiffness dictates lineage commitment. High cell density and extensive cell−cell contact lead to YAP/TAZ inhibition, contributing to contact inhibition of proliferation, whereas low cell density or disrupted cell−cell junctions result in YAP/TAZ activation^19^. Fluid shear stress, particularly relevant for endothelial cells, also regulates YAP/TAZ activity^20^.

The actin cytoskeleton is a central mediator of mechanotransduction, transmitting forces sensed at the cell surface to intracellular signaling pathways^9^. Increased cytoskeletal tension, often driven by Rho GTPases and ROCK, correlates with YAP/TAZ activation^19^. Disruption of the actin cytoskeleton inhibits YAP/TAZ^8^. At adherens junctions, proteins such as E-cadherin and α-catenin sense tension. High tension at low density can lead to conformational changes in α-catenin, recruiting proteins such as TRIP6 that inhibit LATS1/2, thereby activating YAP/TAZ^21^. At high density, reduced tension or specific junctional protein complexes, including NF2, can promote LATS activation and YAP/TAZ inhibition^21^. Focal adhesions, formed by integrins linking the cell to the ECM, are key mechanical sensors. Stiffer substrates promote the formation of larger focal adhesions and stress fibers, activating downstream kinases such as FAK and Src, which can influence YAP/TAZ activity^19^. The specific response of YAP/TAZ to mechanical cues is not universal but varies depending on the cellular context, tissue type and the dimensionality of the environment. This highlights that mechanotransduction is not a simple linear response but is integrated with other cellular signals and structures.

### Noncanonical microenvironmental signals

In addition to canonical Hippo signaling and mechanical cues, YAP/TAZ activity is strongly shaped by various microenvironmental inputs, often via noncanonical pathways or crosstalk with major signaling networks. These include inflammation, hypoxia, metabolic stress and interactions with GPCR, Wnt and RTK pathways (Table 1). YAP/TAZ serve as central integrators of these diverse extracellular signals.

**Table 1:** Noncanonical microenvironmental signals and YAP/TAZ regulation.

Signal category	Specific signals/pathways involved	Key molecular players/mechanisms	Effect on YAP/TAZ activity	Effect on YAP/TAZ localization
**Inflammation**	Pro-inflammatory cytokines (TNF, Prostaglandin E_2_, IL-6)	Induction of YAP/TAZ expression/nuclear translocation	Activation	Nuclear
**Hypoxia**	Low O_2_, HIF-1α	Interaction with HIF-1α, differential phosphorylation (YAP versus TAZ), regulation of angiogenesis, DNA repair, apoptosis, cell cycle	Activation (YAP), inhibition (TAZ)	Nuclear (YAP), cytoplasmic (TAZ)
**Metabolic Stress**	Glucose/energy stress (AMPK, O-GlcNAcylation)	AMPK phosphorylation of YAP/TAZ, AMOTL1 stabilization, O-GlcNAcylation of YAP	Inhibition (low energy), activation (high energy)	Cytoplasmic (low energy), nuclear (high energy)
	Lipid metabolism (mevalonate pathway, statins, Rho GTPases, SCD1, cholesterol, PKA, palmitic acid, TEAD palmitoylation)	Rho GTPase prenylation, SCD1 regulation, cholesterol/PKA/RhoA pathway, palmitic acid-induced MST1/IRF3, TEAD palmitoylation	Activation (mevalonate, SCD1, cholesterol), inhibition (statins, palmitic acid)	Nuclear (mevalonate, SCD1, cholesterol), cytoplasmic (statins, palmitic acid)
	Amino acid metabolism (glutamine transporters, mTOR)	Regulation of glutamine synthetase/transporters, mTOR pathway interaction	Activation	Nuclear
	Oxidative stress (reactive oxygen species, NRF2)	Activation of MST1/2 & LATS1/2 phosphorylation, NRF2 mitigation of YAP1-mediated inflammation	Inhibition	Cytoplasmic
**Crosstalk Signals**	GPCR signaling (Gα12/13, Gαq/11, Gαi/o, Gαs)	Rho GTPase activation, inhibition/activation of LATS1/2 phosphorylation	Activation (Gα12/13, Gαq/11), inhibition (Gαs)	Nuclear (Gα12/13, Gαq/11), cytoplasmic (Gαs)
	Wnt signaling (noncanonical, FZD/ROR, Gα12/13, Rho GTPases, β-catenin complex)	FZD/ROR-Gα12/13-Rho pathway, inhibition of LATS activity, interaction with β-catenin destruction complex, induction of Wnt inhibitors	Activation	Nuclear
	RTK signaling (RAS/PI3K/MAPK, specific RTKs, ERK, AKT)	Downstream pathway activation, direct phosphorylation of YAP/TAZ (for example, Tyr residues), feedback loops	Activation	Nuclear
	TGF-β/Smad signaling	Interaction with SMADs, potential direct regulation or coactivation of targets	Activation	Nuclear
	Notch signaling	Undetermined direct mechanisms, known crosstalk with Hippo pathway	Context dependent	Context dependent

Inflammatory signaling represents a key noncanonical input that modulates YAP/TAZ activity in tissue injury and cancer. Cytokines such as TNF induce YAP/TAZ expression and nuclear translocation^22^, while prostaglandin E_2_ promotes YAP transcription via the EP4–PKA–CREB axis^23^. IL-6 family cytokines activate YAP/TAZ through the gp130–Src–YAP pathway, enhancing nuclear localization—a mechanism crucial for epithelial regeneration during inflammation^24^. These pathways highlight the context-dependent regulatory roles of YAP/TAZ in inflammation.

In parallel, hypoxic conditions within the TME serve as potent modulators of YAP/TAZ signaling. Hypoxia-inducible factors (HIFs), especially HIF-1α, mediate cellular adaptation to low oxygen. YAP/TAZ interact with HIF-1α to drive transcription of genes linked to angiogenesis, survival and proliferation^25^. Notably, hypoxia exerts cell type-specific effects on YAP/TAZ phosphorylation and localization—for example, reducing YAP phosphorylation at S127 (activating it) while increasing TAZ phosphorylation at S89 (inactivating it)^25^.

Metabolic stress, including nutrient deprivation and lipid imbalance, is involved in noncanonical regulation. Energy stress, such as low glucose, activates AMPK, which inhibits YAP/TAZ directly via phosphorylation or indirectly through proteins such as AMOTL1^26^. In contrast, high glucose and nutrient abundance promote YAP/TAZ activation, partly via O-GlcNAcylation. Lipid metabolism is also involved; the mevalonate pathway enhances YAP/TAZ activity by supporting Rho GTPase prenylation, while statins suppress this effect^26^. Stearoyl-CoA desaturase 1 (SCD1), involved in fatty acid synthesis, regulates YAP/TAZ activity^26^. Cholesterol influences TAZ activity through pathways involving PKA and RhoA^27^. Palmitate inhibits YAP activity by inducing MST1 expression via mitochondrial damage and IRF3 activation^27^. TEAD palmitoylation, regulated by cell density, is important for YAP−TEAD binding^27^. Amino acids, particularly glutamine, activate mTOR, which further supports YAP/TAZ activity^26^. Oxidative stress activates the Hippo pathway, leading to YAP/TAZ inhibition^28^. For example, MST1/2 are activated in response to oxidative stress, leading to LATS activation^28^.

In addition to biochemical stressors, receptor-mediated signaling plays a crucial role in modulating YAP/TAZ activity. GPCRs are major upstream regulators: those coupled to Gα12/13 or Gαq/11 activate YAP/TAZ by inhibiting LATS1/2 through Rho GTPases and actin cytoskeleton remodeling^11^, whereas Gαs-coupled GPCRs enhance LATS1/2 activity, thereby inhibiting YAP/TAZ^19^. Wnt signaling—particularly noncanonical branches—activates YAP/TAZ through FZD/ROR receptors and Gα12/13-Rho pathways^29^. In addition, YAP/TAZ crosstalk with canonical Wnt/β-catenin signaling by associating with the β-catenin destruction complex or inducing Wnt antagonists^30^. RTKs modulate YAP/TAZ via RAS/PI3K/MAPK pathways, with ERK and AKT affecting YAP/TAZ expression or phosphorylation. Notably, some RTKs (for example, PDGFR, FGFR, RET and MERTK) directly phosphorylate YAP/TAZ on tyrosine residues, bypassing Hippo pathway regulation^31^. Additional upstream input is provided by transforming growth factor-β (TGF-β)/Smad and Notch signaling pathways^32^.

YAP activation, triggered by diverse cues within the TME, broadly shapes the TME. It promotes ECM remodeling via CAF activation, alters immune cell recruitment and function and enhances angiogenesis—processes that collectively support a protumorigenic and immunosuppressive niche. The following sections explore these effects in detail.

## YAP-driven reprogramming of the TME cell population and characteristics

Beyond driving cancer progression, YAP and TAZ are key regulators of the TME. Their activation in cancer cells reprograms the TME toward an immunologically ‘cold’ microenvironment, characterized by reduced cytotoxic immune infiltration and increased recruitment of suppressive cells^33^. YAP/TAZ also promote fibrosis and ECM stiffening, further limiting immune cell access.

### YAP/TAZ-mediated stromal stiffening during TME remodeling

**Fig. 2:**
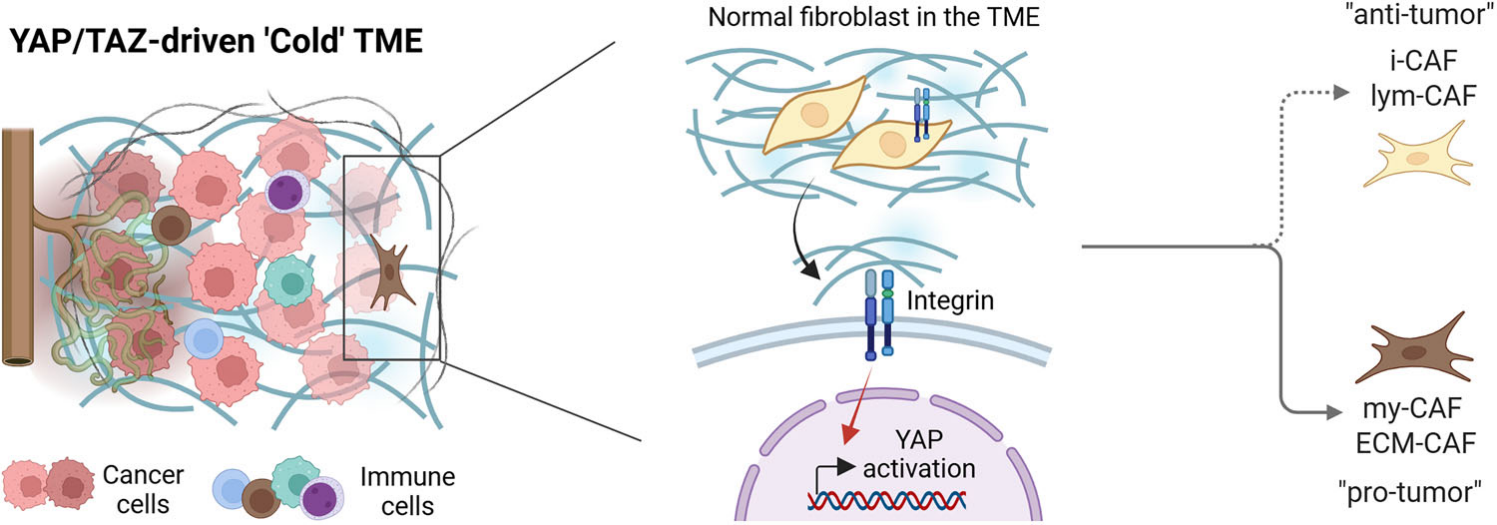
YAP/TAZ-dependent activation of fibroblasts into CAF Subtypes. In the TME, biomechanical (ECM stiffness) cues to activate nuclear YAP/TAZ in the resident fibroblasts, contributing to the CAF population. Activated YAP/TAZ initiate a transcriptional program that skews CAF differentiation toward a myofibroblastic, ECM-producing phenotype (my-CAF/ECM-CAF: α-SMA^high^, high collagen deposition) at the expense of an inflammatory cytokine-secreting i-CAF/lym-CAF fate. my-CAF/ECM-CAFs secrete TGF-β and deposit aligned, crosslinked collagen fibers, increasing stromal stiffness, which reinforces YAP/TAZ nuclear localization via mechanotransduction. The resulting dense ECM barrier excludes immune effectors, establishing an immunosuppressive, ‘cold’ TME. Created in BioRender. Kim, H. (2025) https://BioRender.com/y1gdyu1.

The tumor stroma actively contributes to cancer progression, rather than serving as a passive scaffold. Aggressive solid tumors often exhibit desmoplasia—marked by excessive ECM deposition, stiffness and fibrosis. In this setting, YAP/TAZ function as central regulators by activating and controlling CAFs, thereby influencing ECM dynamics (Fig. 2).

CAFs are a dominant cellular component of the TME in many cancers, arising predominantly from the activated resident fibroblasts, but also from other precursors such as bone marrow-derived cells or through trans-differentiation of epithelial or endothelial cells^34^. Notably, emerging evidence places the YAP/TAZ signaling axis at the core of the fibroblast activation program that generates CAFs, integrating signals from the TME to drive and sustain the activated phenotype. In breast cancer progression, fibroblasts display elevated YAP activity, which distinguishes CAFs from their normal counterparts and correlates with increased contractility and matrix remodeling^35^. This enhanced contractile capacity allows CAFs to exert greater mechanical force on the ECM, promoting further deposition of matrix components and structural remodeling, such as collagen alignment and crosslinking^36^. The resulting increase in ECM stiffness amplifies mechanosignaling, thereby reinforcing YAP/TAZ activation in a feed-forward loop that sustains the myofibroblastic, protumorigenic CAF state. Notably, YAP/TAZ signaling plays a pivotal role in shaping CAF heterogeneity. Their activity is closely linked to the myofibroblastic subtype (my-CAFs) or ECM-CAFs, which are defined by high α-SMA expression, increased contractility and robust ECM deposition and remodeling^37^. In contrast, inflammatory and lymphocyte-associated CAF subsets (i-CAFs and lym-CAFs) exhibit low α-SMA expression and are characterized by high secretion of inflammatory cytokines, such as IL-6 and CXCL family members^38^. These subtypes are often associated with antitumor functions, including enhanced CD8⁺ T cell recruitment and activation. Mechanistically, YAP1 has been shown to suppress the nuclear translocation and transcriptional activity of NF-κB p65, a key driver of the lym-CAF, suggesting that YAP/TAZ may act as a molecular switch controlling the balance between matrix remodeling and immunoregulatory CAFs^37^.

One of the most prominent functions of YAP/TAZ-activated CAFs, particularly the my-CAF/ECM-CAF subtype, is the extensive deposition and remodeling of the ECM^39^. The dense, highly crosslinked and stiff ECM generated through YAP/TAZ-driven CAF activity poses a substantial physical impediment to the infiltration of immune cells including cytotoxic T lymphocyte (CTL) and natural killer (NK) cells into the tumor core^40^. By driving the formation of the fibrotic barrier, YAP/TAZ activity in the TME directly contributes to the establishment of an immunologically ‘cold’ tumor phenotype, shielding cancer cells from immune surveillance and destruction^41^.

Beyond sculpting the physical structure of the stroma, YAP/TAZ activation profoundly influences the immune TME through the release of soluble factors and extracellular vesicles, promoting a tumor-permissive and immune-evasive microenvironment. TGF-β, a potent profibrotic cytokine, activates YAP/TAZ signaling in both fibroblasts and epithelial cells^42^. In turn, YAP/TAZ can enhance components of the TGF-β pathway or amplify its downstream effects, establishing feed-forward loops that sustain fibrosis and promote EMT. TGF-β further contributes to CAF activation and excessive ECM deposition, reinforcing the fibrotic and immunosuppressive nature of the TME.

### YAP/TAZ-orchestrated remodeling of the tumor immune landscape

**Fig. 3:**
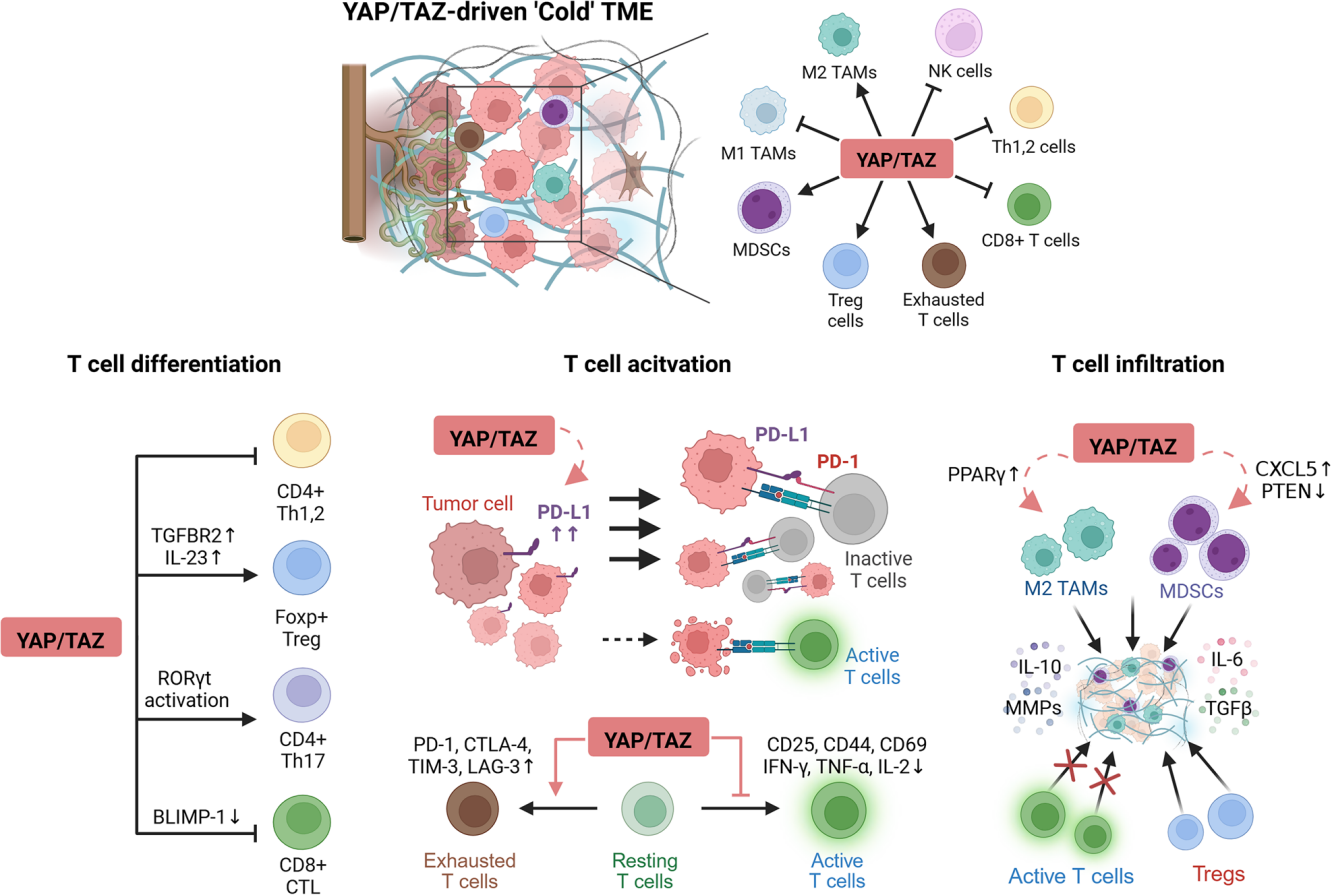
YAP/TAZ-mediated immune remodeling in the TME. Persistent YAP/TAZ signaling skews immune cell composition and function—enhancing recruitment and activation of immunosuppressive populations (M2 TAMs, MDSCs and Tregs) while limiting pro-inflammatory and cytotoxic effectors (M1 TAMs, DCs, NKs and CD8^+^ T cells). Specifically, YAP/TAZ governs T cell activity in the TME; YAP/TAZ not only directs T cell differentiation but also suppresses effector T cell function both directly, by driving exhaustion, and indirectly, by upregulating PD-L1 on tumor cells. Within the stroma, YAP/TAZ elevate CXCL5 and PPARγ while repressing PTEN to recruit M2 TAMs and MDSCs. These myeloid populations secrete IL-10, IL-6, TGF-β and matrix metalloproteinases (MMPs), fortifying the fibrotic barrier that excludes CTLs and NK cells and fosters Treg accumulation. Created in BioRender. Kim, H. (2025) https://BioRender.com/hfw5ye7.

YAP/TAZ signaling is engaged in various immune cells within the TME, where it typically reinforces immunosuppressive environments that facilitate tumor progression (Fig. 3).

Tumor-associated macrophages (TAMs) are highly plastic cells within the TME, polarizing towards either pro-inflammatory, antitumor M1 phenotypes or anti-inflammatory, protumor M2 phenotypes^43^. The TME typically favors the accumulation of M2-like TAMs, which contribute notably to tumor progression through various mechanisms, including suppression of effector T cell responses, promotion of angiogenesis, tissue remodeling and facilitation of metastasis. YAP/TAZ activity in macrophages drives polarization toward an M2-like, tumor-supportive phenotype. TAZ, in particular, induces the transcription factor PPARγ, a key driver of M2 polarization and anti-inflammatory gene expression^44^. Moreover, YAP activation in macrophages enhances the production of cytokines, such as IL-6, which further induces M2-like TAMs and contributes to tumor progression^45,46^. Elevated YAP expression in colon cancer is clinically associated with poor prognosis, probably through its promotion of immunosuppressive M2 polarization^46^; thus, targeting intrinsic YAP/TAZ in TAMs could potentially reprogram them toward an antitumoral M1 phenotype, further enhancing immunotherapy efficacy.

Myeloid-derived suppressor cells (MDSCs) are immature myeloid cells that inhibit T cell responses and promote regulatory T cell (Treg) induction^47^. YAP activation in tumor cells drives both the expansion and recruitment of MDSCs by modulating key cytokines and chemokines, including CXCL5 and potentially granulocyte macrophage-colony stimulating factor through YAP-dependent suppression of PTEN^48^. Notably, elevated YAP1 expression has been positively correlated with increased MDSC infiltration across multiple cancer types.

NK cells are essential components of the innate immune system, endowed with the ability to recognize and eliminate tumor cells without prior sensitization, thereby serving as critical mediators of antitumor immunosurveillance^49^. However, their activity is often suppressed within the TME, which is partially shaped by YAP/TAZ signaling. YAP-enhanced TGF-β signaling directly suppresses NK cell cytotoxicity and IFN-γ production^50,51^. It also promotes their conversion into noncytotoxic ILC1s^52^. High YAP1 expression is negatively associated with the infiltration of activated NK cells in pan-cancer analyses. In ovarian cancer, YAP activation impairs NK cell infiltration by enhancing MDSC recruitment through protein kinase C iota (PRKCι)-dependent signaling^53^.

YAP/TAZ signaling influences the function of dendritic cells (DCs), which are specialized and potent antigen-presenting cells that bridge innate and adaptive immunity. YAP activity promotes an immunosuppressive TME enriched with factors such as TGF-β and immunoregulatory cells including MDSCs and Tregs, thereby impairing DC maturation and functionality^54^. Furthermore, YAP activation in tumor cells suppresses antigen presentation pathways, further compromising effective DC-mediated T cell priming and contributing to immune evasion^55^.

Meanwhile, YAP and TAZ critically regulate T cell activation by modulating expression of the immune checkpoint ligand, programmed death-ligand 1 (PD-L1) on tumor cells^56^. TAZ binds the *PD-L1* promoter via its TBD, driving increased PD-L1 surface expression, delivering an inhibitory signal that suppresses T cell activation and allowing cancer cells to evade immune attack^56^. In addition, intrinsic YAP activation in T cells, potentially triggered by mechanical forces within stiff or dense ECM, directly suppresses key effector functions^57^. YAP activation downregulates T cell activation markers, reduces proliferation and decreases key cytokines, such as IFN-γ, TNF and IL-2 (refs. ^57,58^). Specifically, in CD8^+^ T cells, high YAP1 expression in tumors often correlates negatively with the infiltration of CD8^+^ T cells, suggesting YAP promotes an environment hostile to these effector cells^59^. Consistently, CD4⁺ and CD8⁺ T cells from T cell-specific Yap conditional knockout mice displayed enhanced responsiveness to TCR signaling compared with wild-type counterparts^58^. Moreover, YAP/TAZ have been implicated in driving T cell exhaustion—a dysfunctional state marked by sustained upregulation of inhibitory receptors (PD-1, CTLA-4, TIM-3 and LAG-3) and progressive loss of effector functions^56,60^.

The influence of YAP and TAZ extends to the differentiation of T cells into various functional subsets within the TME. In peripheral blood mononuclear cells from patients with hepatocellular carcinoma, YAP1 is markedly overexpressed and activated, correlating positively with Treg frequency^61^. Mechanistically, YAP augments TGF-β responsiveness by upregulating TGFBR2, thereby driving the differentiation of immunosuppressive FoxP3⁺ Tregs. In murine triple-negative breast cancer cells, TAZ upregulates the production of IL-23, which can promote Treg expansion^62^. TAZ also serves as a coactivator for RORγt, thereby driving pro-inflammatory Th17 differentiation while concurrently inhibiting Treg development^63^. This suggests functional divergence between YAP and TAZ paralogs in shaping CD4⁺ T cell differentiation. In cytotoxic CD8^+^ T cells, YAP suppresses the transcription factor Blimp-1, which is involved in terminal differentiation of CD8^+^ T cells^64^.

In terms of T cell trafficking in the TME, YAP expression in T cells limits their tumor infiltration. In adoptive transfer models, YAP-deficient CD8⁺ T cells showed superior tumor infiltration compared with controls^58^, suggesting YAP as a negative regulator of T cell entry into TME. Moreover, YAP/TAZ indirectly modulate the chemoattractant landscape via other immune components. The YAP−TEAD complex drives CXCL5 and CXCL6 expression to recruit MDSCs, which in turn modulate T cell trafficking and function^65^. YAP/TAZ also regulate CXCL12 expression, which signals through CXCR4 to recruit MDSCs and possibly Tregs, thereby contributing to immune suppression within the TME^66^.

### YAP/TAZ–angiogenesis−hypoxia crosstalk promotes tumor immune escape

Tumor-associated angiogenesis frequently gives rise to abnormal vasculature characterized by disorganized and leaky vessels, poor pericyte coverage and impaired perfusion^67^. YAP and TAZ play central roles in angiogenesis by inducing VEGF and other pro-angiogenic mediators, such as Angiopoietin-2 (Ang-2), thereby supporting both physiological and tumor-associated neovascularization^68^. Notably, VEGF signaling can, in turn, activate YAP/TAZ in endothelial cells via cytoskeletal remodeling and Rho GTPase activation, establishing a positive feedback loop^68^. VEGF signaling may suppress core Hippo kinases LATS1/2 through PI3K/AKT or MEK/ERK cascades downstream of VEGFR2, or alternatively via Rac1–PAK–Merlin inhibition and FAK/Src-mediated blockade of LATS phosphorylation^69^. This interplay establishes YAP/TAZ as pivotal transducers of biochemical and mechanical cues that drive the formation of the dysfunctional tumor vasculature.

**Fig. 4:**
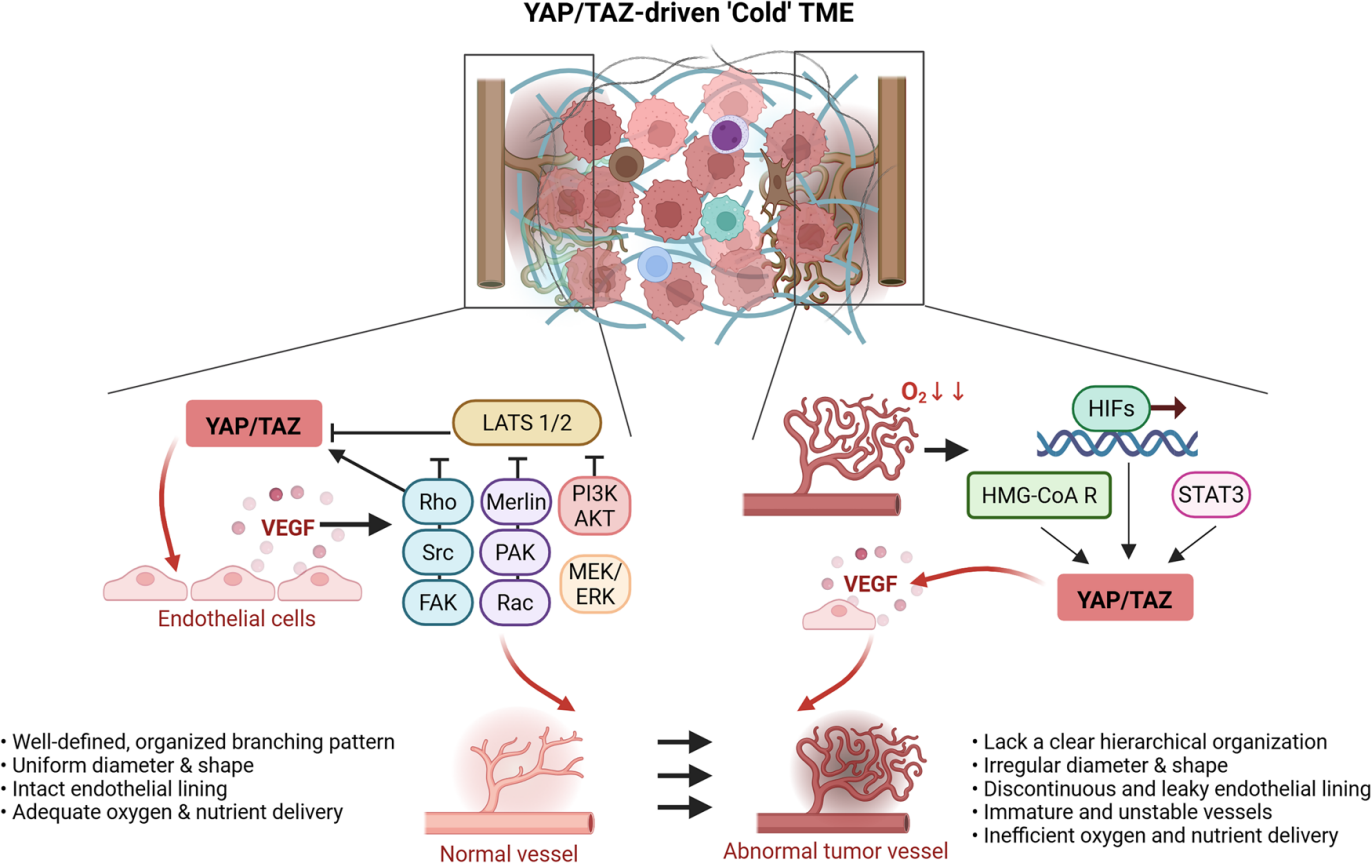
YAP/TAZ–hypoxia–angiogenesis axis in tumor vascular dysfunction. YAP/TAZ promote the expression of pro-angiogenic molecules such as VEGF and Ang-2, leading to the development of a structurally and functionally abnormal tumor vasculature. A positive feedback loop exists where VEGF itself activates YAP/TAZ in endothelial cells (via cytoskeletal changes and Hippo pathway modulation), reinforcing vascular dysfunction. Meanwhile, hypoxia triggers adaptive responses through HIFs and directly activates YAP signaling via multiple pathways (including HIF-dependent GPRC5A or HIF-independent mevalonate pathways). YAP/TAZ can further enhance HIF-1α stability/activity. In endothelial cells, hypoxia-activated STAT3 collaborates with YAP to boost VEGF/Ang-2 production, perpetuating the formation of immature vessels and driving tumor progression. This feed-forward circuit amplifies VEGF production and endothelial YAP/TAZ activity, resulting further vascular abnormalities and hypoxic milieu restrict cytotoxic T cell infiltration and foster an immune-excluded, ‘cold’ TME. Created in BioRender. Kim, H. (2025) https://BioRender.com/fu7lm3a.

The resulting poorly perfused vasculature impairs oxygen and nutrient delivery, leading to tumor hypoxia. Hypoxia activates adaptive transcriptional responses in cancer cells by stabilizing HIFs, which regulate genes involved in angiogenesis, glycolysis, survival and invasion, thereby supporting tumor adaptation to low-oxygen conditions^70^. In hypoxic colorectal cancer cells, GPRC5A—a direct HIF target whose upregulation correlates with poor prognosis—promotes cancer survival under oxygen deprivation by activating YAP signaling^71^. Hypoxia also activates YAP through a HIF-1α-independent mechanism involving the mevalonate pathway and HMG-CoA reductase (HMG-CoA R), driving chemoresistance in hepatocellular carcinoma cells^72^. Notably, YAP/TAZ can interact with HIF-1α to enhance its stability or transcriptional output, thereby amplifying HIF target gene expression, including *VEGF*^73^. These phenomena promote the development of immature, highly proliferative tumor vasculature, cooperating to drive tumor adaptation, progression and malignancy (Fig. 4).

This bidirectional interplay among YAP/TAZ, hypoxia and angiogenesis indicates that targeting a single axis could be insufficient in hypoxic tumors. As both YAP/TAZ and hypoxia can independently or synergistically regulate PD-L1, co-targeting these axes alongside ICB may effectively overcome hypoxia-driven immune evasion.

## Targeting YAP to improve immunotherapy

Given the central role of YAP/TAZ signaling in TME remodeling and immune evasion, various therapeutic strategies have been explored to inhibit aberrant YAP/TAZ activation in both preclinical and clinical studies (Table 2), suggesting potential synergy with immunotherapy.

**Table 2:** YAP-targeted therapeutic agents.

Strategies	Agent	Specific target	Mechanisms and key notes
Direct YAP/TAZ inhibitors	VP	YAP–TEAD binding	First identified YAP–TEAD inhibitor, requires high μM doses and has off-target effects; phase 2 (NCT06381154; combined with anti-PD-1 Pembrolizumab)
	VGLL4-mimetic peptides	YAP–TEAD binding	Compete with YAP/TAZ for TEAD binding, harnesses endogenous corepressor motif, delivery and stability remain challenges
	YAP TBD-derived peptides	YAP–TEAD binding	Decoy peptides that bind TEAD, blocking YAP/TAZ assembly, systemic delivery limitations
	Pan-TEAD PPI disruptor (IAG933, SW-682)	YAP–TEAD binding	Covalent/noncovalent binders of TEAD catalytic cysteine in lipid-binding pocket; orally bioavailable; evicts YAP from chromatin and suppresses TEAD-driven transcription; IAG933: phase 1 (NCT04857372); SW-682: phase 1a/1b (NCT06251310)
	TEAD palmitoylation inhibitors (VT3989, IK-930)	Allosteric TEAD inhibitor	Covalent/noncovalent binders of TEAD catalytic cysteine in lipid-binding pocket; VT3989: phase 1 (NCT04665206; combined with anti-PD-1 Nivolumab + anti-CTLA-4 Ipilimumab); IK-930: phase 1 (NCT05228015)
Upstream modulators of YAP/TAZ	Compound C19	LATS1/2 kinases	Promotes YAP degradation via Inducing LATS phosphorylation/activation
	NUAK2 inhibitors	NUAK family kinase 2	Disrupt NUAK2–YAP positive feedback → enhance cytoplasmic YAP retention and degradation
	BRD4 (BET) inhibitors	BRD4	Block YAP/TAZ–BRD4 interaction → reduce enhancer recruitment and target gene expression (*CTGF/CYR61*)
	STAT3 inhibitors	STAT3 signaling	Block YAP activation and modulate immunosuppression
Inhibitors of downstream effectors	Bemcentinib (BGB324)	AXL receptor tyrosine kinase	First-in-class AXL inhibitor, antifibrotic effects (IPF trials), potential to modulate fibrotic TME in cancer
	Pamrevlumab (FG-3019)	CTGF	Monoclonal antibody neutralizes CTGF, reduces fibrosis and stromal activation, approved in fibrotic diseases

### Molecular strategies targeting YAP/TAZ for TME modulation

Current direct-targeting strategies against YAP/TAZ aim either to disrupt their functional interaction with TEAD transcription factors or to disable their transcriptional activity by modifying transactivation domains.

Verteporfin (VP), a benzoporphyrin derivative clinically approved as a photosensitizer for photodynamic therapy in age-related macular degeneration, was the first compound identified to inhibit YAP−TEAD binding^74^. Structural studies suggest that VP binds directly to YAP, while functional evidence in uveal melanoma cells indicates it also promotes lysosome-dependent degradation of YAP^74^. Preclinical studies have shown that VP can inhibit YAP-dependent processes relevant to the TME, including reducing TAM recruitment and suppressing tumor angiogenesis^74^. In a murine model of cholangiocarcinoma driven by YAP/AKT, VP treatment reduced tumor burden and modulated the immune microenvironment, increasing the ratio of antitumor M1 TAMs to protumor M2 TAMs and boosting the activation of CD8^+^ T cells^75^.

Peptides designed to mimic YAP/TAZ binding interfaces represent another therapeutic modality. For instance, VGLL4-mimicking peptides exploit the inherent competitive interaction between YAP/TAZ and the transcriptional corepressor VGLL4 for TEAD binding, effectively harnessing its role as a natural antagonist of YAP−TEAD-mediated transcription^76^. In addition, peptides derived directly from the TBD of YAP^77^ or dominant-negative peptides/proteins (pTEADi/TEADi)^78^ can act as competitive inhibitors. When overexpressed, these TBD constructs function as decoys that bind and sequester TEAD proteins, thereby blocking endogenous YAP and TAZ from assembling into active transcriptional complexes. However, delivery and stability issues limit the systemic application of peptide-based approaches.

Meanwhile, considerable efforts are underway to develop more potent and orally bioavailable small-molecule inhibitors of the YAP/TAZ−TEAD interaction. IAG933 represents a first-in-class example of a potent, direct pan-TEAD PPI disruptor suitable for clinical testing, developed through optimization of a YAP peptide pharmacophore^79^. IAG933 functions by directly competing with YAP/TAZ for binding to TEADs, leading to the eviction of YAP from chromatin, suppression of TEAD-driven transcription and induction of cancer cell death. Another small-molecule inhibitor, GNE-7883, also functions as an allosteric pan-TEAD inhibitor blocking YAP/TAZ interaction. The discovery that TEAD proteins undergo S-palmitoylation at a conserved cysteine within a central lipid-binding pocket has revealed a defined and druggable target^80,81^. Numerous small molecules targeting this pocket, either through noncovalent binding or covalent modification of the catalytic cysteine, function as allosteric inhibitors of TEAD activity^82,83^. These compounds effectively disrupt the YAP/TAZ−TEAD interaction, consequently suppressing downstream transcriptional activity.

Targeting upstream regulatory pathways offers an alternative approach to indirectly suppress pathological YAP/TAZ signaling. Since YAP/TAZ are mechanoresponsive, agents that disrupt cytoskeletal tension—such as statins (simvastatin, atorvastatin), which inhibit HMG-CoA reductase and thereby impair Rho GTPase prenylation—reduce YAP/TAZ nuclear localization^84^. The core Hippo kinases LATS1/2 phosphorylate and inactivate YAP/TAZ, while compound C19 promotes LATS phosphorylation and YAP degradation, suppressing tumor growth in vivo^85^. Conversely, NUAK2 sustains YAP/TAZ by inhibiting LATS activity and is upregulated by YAP/TAZ–AP-1 in a feed-forward loop. Genetic or pharmacologic NUAK2 inhibition restores YAP/TAZ cytoplasmic sequestration and diminishes TEAD transcription^86^. Epigenetic modulation via BET inhibitors—targeting BRD4, a cofactor for YAP/TAZ−TEAD complexes—suppresses downstream targets (connective tissue growth factor (CTGF) and CYR61) even under YAP overexpression^87^. In addition, STAT3—a critical YAP/TAZ activator in tumor endothelium—represents an attractive dual-purpose target as its inhibition may impair both angiogenesis and immunosuppressive YAP/TAZ signaling^88^.

An additional therapeutic strategy aims to inhibit key downstream targets responsible for mediating the tumor-promoting effects of YAP/TAZ. The RTK AXL—upregulated by YAP/TAZ and linked to metastasis, immune evasion and therapy resistance—can be inhibited by the oral small molecule bemcentinib (BGB324), which reverses EMT, restores an immunostimulatory TME and sensitizes tumors to chemotherapy^89^. Similarly, CTGF—a YAP/TAZ–TEAD target that drives fibrosis and angiogenesis—is neutralized by the monoclonal antibody pamrevlumab (FG-3019), currently being tested in oncology to modulate tumor stroma^90^. These strategies may be more effective than upstream kinase or GPCR targeting, which is limited by pathway redundancy, and may spare normal YAP/TAZ functions disrupted by direct TEAD inhibition.

### Combining YAP pathway targeting with immunotherapy: preclinical and clinical strategies

The ability of YAP/TAZ inhibitors to reprogram multiple components of the TME provides a compelling rationale for their combination with conventional cancer therapies. By reducing immunosuppression, alleviating fibrosis and normalizing tumor vasculature, YAP/TAZ inhibition may help overcome therapeutic resistance and synergize with immunotherapy and other targeted treatments.

#### Combination of YAP targeting with ICB

YAP/TAZ-driven immunosuppressive TME is a major barrier to effective immunotherapy^91^. YAP/TAZ-induced PD-L1 expression supports combining their inhibition with immune checkpoint blockades (ICBs)^6^. Combining YAP/TAZ pathway inhibitors with ICBs offers several potential synergistic mechanisms. First, inhibition of YAP/TAZ is expected to reduce the transcription and surface expression of PD-L1 on tumor cells, thereby limiting its engagement with PD-1 and alleviating T cell suppression, thereby sensitizing the tumor. Importantly, PD-L1 is also expressed on various stromal and immune cells in the TME—such as MDSCs, TAMs and CAFs—where it contributes to immune evasion. Thus, YAP/TAZ inhibition may reprogram multiple immunosuppressive cell types, broadening its impact on the tumor immune landscape^56^. In addition, by modulating the recruitment and function of MDSCs, Tregs and M2-polarized TAMs, YAP/TAZ inhibition alleviates key immunosuppressive pressures and enhances the activity of effector T cells stimulated by ICBs. Finally, YAP inhibition reduces CAF activation and ECM deposition, facilitating CTL infiltration by lowering stromal barriers.

Emerging preclinical evidence supports the therapeutic benefit of combining YAP pathway inhibition with ICBs. For example, combining VP with anti-PD-1 therapy showed synergistic antitumoral effects in a murine cholangiocarcinoma model, linked to favorable immune modulation within the TME^92^. Similarly, indirect targeting of YAP via STAT3 inhibition improved survival in pancreatic ductal adenocarcinoma models when paired with ICB^93^. These findings call for direct evaluation of synergy between YAP/TEAD inhibitors and ICBs.

#### Combining YAP suppression with CAR-T cell therapy

Considering the role of YAP/TAZ in forming physical and immunosuppressive barriers, YAP pathway inhibition could enhance chimeric antigen receptor (CAR)-T cell therapy by remodeling the TME, sensitizing tumor cells to immune killing and improving CAR-T function. While direct evaluation of pharmacological YAP inhibitors with CAR-T cells remains limited, emerging evidence suggests their potential to augment efficacy.

YAP-suppressed CAR-T cells exhibit enhanced activation upon antigen encounter, improved proliferation and persistence, better resistance to exhaustion and more effective tumor infiltration and killing, implying a cell-intrinsic approach to boosting CAR-T cell fitness and improving antitumor responses^58^. Another study investigated the involvement of YAP activity in neuroblastoma resistance to anti-GD2 immunotherapy^94^. Genetic YAP inhibition in tumor cells increased the surface expression of the target antigen GD2 and markedly sensitized these tumors to killing by anti-GD2 antibody-dependent cellular cytotoxicity mediated by γδ T cells. This shows that YAP inhibition can modulate tumor antigen expression and overcome YAP-mediated resistance in the TME. Furthermore, YAP activation induces softness in both malignant T cells and CTLs^95^. Malignant T cells were more sensitive to YAP inhibitors than CTLs, potentially due to lower expression of the MDR1 drug transporter in malignant cells. Moderate YAP inhibition increased malignant T cell stiffness, enhancing their susceptibility to CTL killing while preserving CTL function. This suggests a potential therapeutic window for using YAP inhibitors to enhance T cell-mediated killing in T cell malignancies.

#### Combining YAP targeting with tumor vaccines and other therapeutic agents

Tumor vaccines aim to elicit or boost endogenous tumor-specific T cell responses capable of recognizing and eliminating cancer cells by delivering tumor-associated or tumor-specific antigens^96^. However, their efficacy is often limited by low tumor antigen immunogenicity and a profoundly immunosuppressive TME, which impairs the priming, expansion, trafficking and function of vaccine-induced T cells. Given that YAP/TAZ activity can influence antigen presentation pathways or the differentiation state of tumor cells, inhibiting YAP/TAZ could potentially enhance vaccine efficacy. YAP/TAZ inhibition may also enhance the antitumor activity of vaccine-induced T cells by relieving TME-mediated immunosuppression.

Preclinical evidence supporting this concept includes the finding that YAP ablation in pancreatic cancer models reversed key immunosuppressive features, blocking MDSC recruitment, favoring antitumor macrophage polarization and T cell reactivation^97^. A recent study developed attenuated yet immunogenically potentiated tumor extracellular vesicles (AI-TEVs) as a personalized cancer vaccine platform^98^. AI-TEVs were derived from tumor cells treated with VP and autophagy inhibitors. Dual inhibition reduced the malignant properties of the TEVs while enriching them with tumor antigens and adjuvants, potentially due to ICD induction. Vaccination with AI-TEVs inhibited tumor growth prophylactically and in mouse models, inducing tumor-specific and durable immune memory. These findings provide direct evidence that pharmacological YAP suppression enhances vaccine efficacy. Similar findings in pancreatic cancer models further support this concept^97^. Another study linked YAP signaling to the protective effects of *Bifidobacterium adolescentis* against colorectal tumorigenesis, demonstrating that it promotes the production of the extracellular matrix component decorin through a TLR2/YAP-dependent pathway and modulates macrophage polarization^99^. Furthermore, vaccines targeting TWIST1—a YAP-linked EMT transcription factor—elicited robust CD8⁺ T cell responses in mesothelioma and breast cancer models^100^. Collectively, YAP-associated remodeling of the immune landscape holds potential to synergize with vaccine strategies.

In addition, suppression of YAP/TAZ signaling holds strong potential to enhance next-generation antibody therapeutics. Bispecific antibodies, which simultaneously target tumor antigens and immune effectors or checkpoints, often underperform in immunosuppressive TMEs. YAP/TAZ inhibition remodels this environment by reducing matrix stiffness, dampening immunosuppressive cytokines and facilitating immune cell infiltration, thereby restoring bispecific antibody activity. Similarly, YAP/TAZ signaling promotes resistance to antibody–drug conjugates via EMT and metabolic rewiring, both of which impair payload delivery. Cotreatment with YAP/TAZ inhibitors can overcome both microenvironmental and intrinsic resistance mechanisms, enhancing antibody–drug conjugate efficacy.

#### Clinical trials of YAP inhibition combined with immunotherapy

YAP/Hippo pathway modulators are being clinically investigated in combination with immunotherapies, particularly for cancers marked by aberrant YAP/TAZ activity and poor immune responsiveness (Table 3). Mesothelioma and pancreatic cancer have emerged as initial targets in clinical trials, given their strong association with YAP/TAZ dysregulation and the limited efficacy of existing immunotherapeutic approaches.

**Table 3:** Clinical trials combining YAP pathway modulators with immunotherapy.

Trial ID	YAP/Hippo modulator (mechanism)	Immunotherapy agent(s)	Cancer type(s)	Phase/key objectives
NCT04665206	VT3989 (oral YAP/TEAD inhibitor)	Nivolumab (anti-PD-1) + Ipilimumab (anti-CTLA-4)	Malignant mesothelioma (unresectable/metastatic)	Phase 1/safety, tolerability, preliminary efficacy of combination
NCT06381154	VP (IV, photodynamic therapy/YAP inhibitor)	Pembrolizumab (anti-PD-1)	Pancreatic ductal adenocarcinoma (unresectable/metastatic)	Phase 2/safety, overall response rate, duration of response, progression-free survival
NCT06251310	SW-682 (oral TEAD inhibitor)	Appropriate combination therapy (immunotherapy partner unspecified yet)	Advanced solid tumors	Phase 1b/safety, tolerability, preliminary efficacy of combination

The phase 2 trial NCT06381154 is investigating VP-mediated photodynamic priming combined with Pembrolizumab (anti-PD-1) and standard chemotherapy in patients with unresectable, locally advanced or metastatic pancreatic cancer. The trial is ongoing, and its results may clarify the immunomodulatory potential of VP in pancreatic cancer, a treatment-resistant cancer with limited ICB efficacy.

In Part 2, Cohort 4 of the phase 1b study NCT06251310 (SW-682 combination cohort), the oral TEAD inhibitor, SW-682 is being evaluated in combination with conventional systemic anticancer therapy in patients with mesothelioma and other advanced solid tumors. Another TEAD inhibitor, VT3989, an allosteric modulator, is currently being tested in combination with the immune‑checkpoint inhibitors, nivolumab (anti‑PD‑1) and ipilimumab (anti‑CTLA‑4), in solid tumors harboring Hippo pathway alterations such as NF2 loss or YAP/TAZ activation, including malignant pleural mesothelioma (NCT04665206).

FAK inhibitors, which modulate YAP activity through mechanotransduction, are being explored in combination regimens, potentially with immunotherapies, supported by strong preclinical data. Additionally, BET inhibitors (for example, ZEN003694), which can indirectly affect YAP/TAZ activity via BRD4 inhibition, are being clinically tested in combination with ICBs (Nivolumab ± Ipilimumab) in solid tumors (for example, NCT04840589).

So far, the direct application of the YAP/TAZ signaling pathway in CAR-T cell therapy or vaccine development remains limited. Most studies have explored YAP/TAZ as an adjunctive target to overcome immune suppression in the TME. However, if therapeutic strategies directly targeting YAP/TAZ are developed, they could represent a novel and effective approach to enhance the efficacy of CAR-T cells or therapeutic cancer vaccines by modulating immune regulation at the transcriptional level.

## Challenges and future directions

Despite the promise of YAP/TAZ inhibition in immunotherapy, several challenges hinder clinical translation. A major concern is how to suppress tumor-specific YAP/TAZ activity without disrupting their physiological roles in tissue homeostasis and immune regulation. Since YAP/TAZ are not exclusively oncogenic, therapies must balance tumor suppression with immune preservation and minimal systemic toxicity. Selective stromal inhibition or localized delivery using tumor-targeted nanoparticles offers a promising strategy to enhance precision and minimize off-target effects. Moreover, the inherent plasticity and redundancy of signaling networks in cancer cells pose substantial risks for acquired resistance to YAP/TAZ inhibition. Tumors may undergo adaptive metabolic reprogramming or undergo structural and biomechanical changes within the ECM, effectively sustaining tumor growth and immune evasion independent of YAP/TAZ activity. To overcome such resistance mechanisms, it will be critical to employ combination strategies targeting multiple complementary signaling pathways, exploiting synthetic vulnerabilities or modulating specific aspects of TME remodeling.

The success of YAP/TAZ-targeted therapies also relies on robust biomarkers. Proposed candidates include YAP/TAZ nuclear localization, target gene expression, Hippo pathway mutations and immune features such as PD-L1 expression and TME composition. However, the pleiotropic and context-dependent nature of YAP/TAZ signaling means that high expression does not necessarily indicate functional dependency. Effective biomarker development will require integrative approaches that capture dynamic, context-specific pathway activity.

Single-cell and spatial transcriptomics provide powerful tools to resolve these complexities. While single-cell RNA-sequencing offers high-resolution profiling of YAP/TAZ activity at the individual cell level, spatial transcriptomics preserves tissue architecture, enabling analysis of mechanical cues and intercellular signaling. These tools are well suited to dissecting the mechanoresponsive and non-cell-autonomous behavior of YAP/TAZ. Combining these with advanced modalities such as CITE-sequencing and scATAC-sequencing will facilitate spatially informed functional maps of YAP/TAZ activity across the TME.

In summary, targeting YAP/TAZ holds strong therapeutic promise, but success hinges on overcoming challenges in specificity, delivery and pathway complexity. A multidisciplinary approach integrating mechanistic insights, spatial omics and rational drug design will be key to advancing YAP/TAZ-based immunotherapies.
